# Non-Apical Membrane Antigen 1 (AMA1) IgGs from Malian Children Interfere with Functional Activity of AMA1 IgGs as Judged by Growth Inhibition Assay

**DOI:** 10.1371/journal.pone.0020947

**Published:** 2011-06-13

**Authors:** Kazutoyo Miura, Suwani Perera, Sarah Brockley, Hong Zhou, Joan A. Aebig, Samuel E. Moretz, Louis H. Miller, Ogobara K. Doumbo, Issaka Sagara, Alassane Dicko, Ruth D. Ellis, Carole A. Long

**Affiliations:** 1 Laboratory of Malaria Immunology and Vaccinology, National Institute of Allergy and Infectious Diseases, National Institutes of Health, Rockville, Maryland, United States of America; 2 Laboratory of Malaria and Vector Research, National Institute of Allergy and Infectious Diseases, National Institutes of Health, Rockville, Maryland, United States of America; 3 Malaria Research and Training Center, Faculty of Medicine Pharmacy and Dentistry, University of Bamako, Bamako, Mali; London School of Hygiene and Tropical Medicine, United Kingdom

## Abstract

**Background:**

Apical membrane antigen 1 (AMA1) is one of the best-studied blood-stage malaria vaccine candidates. When an AMA1 vaccine was tested in a malaria naïve population, it induced functionally active antibodies judged by Growth Inhibition Assay (GIA). However, the same vaccine failed to induce higher growth-inhibitory activity in adults living in a malaria endemic area. Vaccination did induce functionally active antibodies in malaria-exposed children with less than 20% inhibition in GIA at baseline, but not in children with more than that level of baseline inhibition.

**Methods:**

Total IgGs were purified from plasmas collected from the pediatric trial before and after immunization and pools of total IgGs were made. Another set of total IgGs was purified from U.S. adults immunized with AMA1 (US-total IgG). From these total IgGs, AMA1-specific and non-AMA1 IgGs were affinity purified and the functional activity of these IgGs was evaluated by GIA. Competition ELISA was performed with the U.S.-total IgG and non-AMA1 IgGs from malaria-exposed children.

**Results:**

AMA1-specific IgGs from malaria-exposed children and U.S. vaccinees showed similar growth-inhibitory activity at the same concentrations. When mixed with U.S.-total IgG, non-AMA1 IgGs from children showed an interference effect in GIA. Interestingly, the interference effect was higher with non-AMA1 IgGs from higher titer pools. The non-AMA1 IgGs did not compete with anti-AMA1 antibody in U.S.-total IgG in the competition ELISA.

**Conclusion:**

Children living in a malaria endemic area have a fraction of IgGs that interferes with the biological activity of anti-AMA1 antibody as judged by GIA. While the mechanism of interference is not resolved in this study, these results suggest it is not caused by direct competition between non-AMA1 IgG and AMA1 protein. This study indicates that anti-malaria IgGs induced by natural exposure may interfere with the biological effect of antibody induced by an AMA1-based vaccine in the target population.

## Introduction

WHO estimates there were 243 million malaria cases and 0.9 million deaths in 2008; the vast majority of deaths occurred in African children less than 5-years old due to *Plasmodium falciparum*, which is the most virulent species of human malaria [1]. While the protective mechanisms remain to be elucidated, a passive transfer study has shown the importance of gamma-globulin against blood-stages of *P. falciparum* [2]. To control and eventually eradicate malaria, an effective vaccine is considered to be needed, in addition to the existing tools, such as drugs, insecticides, etc. [3].

Apical membrane antigen 1 (AMA1) is the one of the best-studied blood-stage vaccine candidates and it is an essential protein for parasite invasion of an erythrocyte [4]. The invasion process is complicated (i.e., initial attachment, reorientation, tight junction formation and internalization), and different studies have suggested different roles for AMA1: binding to erythrocytes [5]–[7], reorientation [8], or internalization [9]. In addition to erythrocyte invasion, a recent study suggests that AMA1 is involved in sporozoite invasion of hepatocytes [10]. These results indicate the AMA1 protein may have multiple roles. Many studies have shown that AMA1 vaccination can induce protective immunity in animal models (reviewed in [11]) and in an *Aotus* monkey challenge model (the monkeys were challenged with *P. falciparum*). In the monkey challenge model, anti-AMA1 antibody levels induced by a vaccine before challenge have shown to correlate with protection [12], [13]. In addition to the animal data, many, but not all, epidemiological studies suggest a high level of AMA1 antibodies is associated with a reduced risk of malaria [11]. Based on these findings, multiple AMA1 Phase 1 trials [14]–[24] and a Phase 2 field trial [25] have been conducted and published. However, to date no significant protective effects have been shown in the target population of African children.

An in vitro parasite Growth Inhibition Assay (GIA; also referred to as the Invasion Inhibition Assay, IIA) is one of the few widely-used biological assays that can measure the functional activity of antibodies against blood-stage malaria. While it is still controversial whether the activity measured by the GIA (IIA) reflects protective immunity induced by a vaccine, the assay has been used in preclinical and clinical studies as one of the immunological readouts. Not only anti-AMA1 antibodies induced by malaria infection [26], [27], but also antibodies induced by AMA1 immunization in malaria naïve individuals show growth-inhibitory activity in the *in vitro* GIA [14], [16], [19], [21]–[23].

In contrast, while the same AMA1 vaccine increased the anti-AMA1 antibody levels when it was administered to adults who lived in a malaria endemic area, the vaccine did not change the parasite growth-inhibitory activity [15]. In a previous study, we purified total IgGs from sera collected in an epidemiological study in Mali (majority of the sera were collected from adults) and separated the IgGs by affinity chromatography into an AMA1-binding fraction (AMA1-specific) or an IgG fraction that does not bind to AMA1 (non-AMA1 IgG). Previously we have shown that the non-AMA1 IgG, more specifically a fraction of the non-AMA1 IgG which can bind to malaria extract, reduced the functional activity of the AMA1 antibodies from U.S. vaccinees [27]. Interference by the non-AMA1 IgG induced by malaria infections likely explains the reason why the AMA1 vaccine did not induce higher growth-inhibitory activity in the Malian adults in the vaccine trial. Because limited volumes of sera were collected from children in the epidemiological study, we could not investigate whether there was such “interfering” IgG in the children, who are the main target population of the blood-stage vaccine and who have less previous exposure to malaria.

Our recent Phase 2 trial in Malian children showed that there was a small, but significant, increase of growth-inhibitory activity when data from all AMA1-immunized children was analyzed (the GIA was performed with total IgGs in the study) [28]. The level of increase in the activity was not different from the level observed in U.S. adults who were immunized with the same vaccine formulation [28]. In the U.S. vaccine trial, all of the volunteers showed negligible levels of inhibition before vaccination, and after immunization the growth-inhibitory activity of total IgG was a function of anti-AMA1 titer. However, in the Malian children, the increase of growth-inhibitory activity was observed only in AMA1-immunized children who had less than 20% inhibition before vaccination. None of the children who had more than 20% inhibition before vaccination (14/89 children in the AMA1-immunized group) showed more than a 20% increase, while antibody levels measured by ELISA increased in most of the children. On the other hand, out of the remaining 75 children with less than 20% inhibition before vaccination, 25 (33%) children had more than a 20% increase after vaccination. In the present study we wished to investigate the effect of non-AMA1 IgGs in the children to determine whether similar interference effects could be identified. Therefore, we made multiple pools of total IgG, separated them into AMA1-specific and non-AMA1 IgGs, and tested them by GIA. The non-AMA1 IgGs showed an interference effect on growth-inhibitory activity.

## Materials and Methods

### Clinical trials and blood collections

The details of the U.S. adult Phase 1 trial [16] and the Phase 2 trial in Malian children [25] have been supplied elsewhere (NCT00344539 and NCT00341250). In brief, adults 18–45 years of age were enrolled in the U.S. trial and they were immunized on Days 0, 28 and 56 with 20 or 80 µg of AMA1-C1 (a mixture of the recombinant AMA1-FVO and AMA1-3D7 proteins) formulated on Alhydrogel® and mixed with CPG 7909. Plasma samples from individuals with high levels of anti-AMA1 antibody (as determined by ELISA) on Day 70 were collected 3 months after the final vaccination. For the Mali trial, Malian children 2–3 years old were enrolled and immunized on Days 0 and 28 either with 80 µg of AMA1-C1 on Alhydrogel® or a comparator vaccine (Hiberix®). Blood samples were collected on Days 0 and 42. Both trials were conducted under Investigational New Drug Applications reviewed by the U.S. Food and Drug Administration. The U.S. Phase 1 trial was reviewed and approved by the Institutional Review Boards (IRB) of the National Institute of Allergy and Infectious Diseases (NIAID), National Institutes of Health (NIH) and by the University of Rochester Research Subjects Review Board. Written informed consent was obtained from all volunteers. The Mali Phase 2 trial was reviewed and approved by the IRB of NIAID at NIH and by the Ethics Committee of the Faculty of Medicine, Pharmacy and Dentistry, University of Bamako. Community consent was obtained at a meeting with village leaders, heads of families, and other community members prior to the start of the Phase 2 study. Individual informed consent was then obtained after oral translation of the consent form into the local language. Understanding of the contents of the consent was confirmed by means of a multiple choice questionnaire. Parents or guardians unable to read placed an imprint of his/her finger in place of a signature; an independent witness also signed all consent forms.

### Total, AMA1-specific and non-AMA1 IgG preparations

Total IgGs were purified from individual plasma samples from the U.S. trial (n = 5) and normal U.S. sera (n = 2) using Protein G columns as described previously [14]. Similarly, total IgG was prepared from each plasma sample collected on Days 0 and 42 from the Mali Phase 2 trial. All of the total IgGs were dialyzed against RPMI 1640 and concentrated to 40 mg/ml. Because the volume of the total IgG from each individual Malian child was not enough to perform AMA1-specific IgG purification, the total IgG samples were ranked based on their anti-AMA1(3D7) antibody level as judged by ELISA and were divided into 3 or 4 groups to make pooled IgGs at each time point as follows: For Day 0 IgGs (regardless of immunization groups), D0-1, D0-2, D0-3 and D0-4; for Day 42 IgGs from Hiberix®-vaccinated children, Hib-1, Hib-2 and Hib-3; for Day 42 IgGs from AMA1-vaccinated children, AMA1-1, AMA1-2 and AMA1-3. The number of individual total IgGs used to make each total IgG pool and the antibody level of the pool is shown in Table 1. Although all of the anti-malarial antibody in Day 0 IgGs and Day 42 IgGs from the Hiberix group was induced by natural infection, it is possible that vaccination with Hiberix® and/or the timing of blood collection during the malaria transmission season might alter the immune response. Therefore, we made separate pooled IgGs from Day 0 IgGs and Day 42 IgGs from Hiberix® group in this study.

**Table 1:** Anti-AMA1 antibody level [mg/ml] in test IgGs (40 mg/ml).

		AMA1-3D7^b^			AMA1-FVO^c^		
	n^a^	Total IgG pool	Non-AMA1 (3D7) IgG		Total IgG pool	Non-AMA1 (FVO) IgG	
D0-1	93	2^d^	2^d^	(ND^g^)^f^	2^d^	2^d^	(ND^g^)
D0-2	38	6	N/A^e^		8	2^d^	(ND^g^)
D0-3	46	35	N/A^e^		63	2^d^	(ND^g^)
D0-4	27	439	13	(3)	675	19	3
Hib-1	47	2^d^	2^d^	(ND^g^)	2^d^	2^d^	(ND^g^)
Hib-2	33	10	2^d^	(ND^g^)	16	2^d^	(ND^g^)
Hib-3	35	246	7	(3)	334	8	(2)
AMA1-1	31	66	4	(6)	100	9	(9)
AMA1-2	41	260	16	(6)	354	17	(5)
AMA1-3	41	751	24	(3)	1,050	47	(4)

a Number of individual IgGs to make total IgG pool.

b Antibody level [µg/ml] to AMA1(3D7) ELISA plate.

c Antibody level [µg/ml] to AMA1(FVO) ELISA plate.

d Less than the minimal detection level of antibody.

e N/A, No sample available.

f Numbers in parentheses, percent relative to the total IgG.

g ND, Percent cannot be determined.

From these U.S. total IgG and Mali total IgG pools, AMA1(3D7) or AMA1(FVO)-specific IgGs and non-AMA1(3D7) or non-AMA1(FVO) IgGs were prepared individually using AMA1(3D7) or AMA1(FVO) protein immobilized on Sepharose 4 Fast Flow columns as described previously [27]. During the affinity purification process for each total IgG, the flow-through fraction was reloaded to the same AMA1-immobilized column three times to increase the efficacy of separation. Both AMA1-specific IgGs and non-AMA1 IgGs were dialyzed against RPMI 1640 and concentrated to 150∼300 µl (AMA1-specific IgGs) or 40 mg/ml (non-AMA1 IgGs) of final product. Because of the limitations of volumes available, AMA1(3D7)-specific/non-AMA1(3D7) IgGs were not prepared from D0-2 or D0-3 total IgG pools.

### ELISA

The standardized methodology for performing the ELISA has been described previously [29]. The absorbance of each test sample was converted into ELISA units using a standard curve generated by serially diluting the standard in the same plate. The ELISA units of each sample were then converted to µg/ml using a conversion factor as described elsewhere [30]. The minimal detection level of the AMA1 antibody in this study was 4 µg/ml, and all responses below that limit of detection were assigned a value of 2 µg/ml for the analysis.

For the competition ELISA, a fixed amount of total IgG from a U.S. vaccinee (designated as US-total IgG), which gives approximately an O.D. value of 3 (approximately 30–40 µg/ml of total IgG in ELISA well), was mixed with 2-fold dilutions of non-AMA1(3D7) IgGs from Malian children (ranging from 2 to 133 µg/ml in ELISA well). The mixtures were tested by ELISA using an AMA1(3D7) or AMA1(FVO)-coated plate using the standard ELISA procedure, and the direct O.D. value was used as a final readout, instead of ELISA units.

### GIA

The standard methodology for the GIA has been described previously [14]. The assay was performed with purified IgGs at indicated concentrations against the 3D7 or FVO strain of *P. falciparum* parasites.

For the mixture GIA experiment, 4 mg/ml of non-AMA1(3D7) or non-AMA1(FVO) IgGs were mixed with US-total IgG. The concentration of US-total IgG was determined at which the IgG was expected to give ∼60% inhibition in the final mixture in the standard GIA. The growth-inhibitory activity of mixtures was determined using the standard GIA.

### Statistical analysis

The correlation between the two data sets (e.g., anti-AMA1 antibody level and % inhibition in GIA, etc.) was tested by a Spearman rank correlation test. Best-fit formulations of the GIA data were calculated using logarithm-transformed antibody levels. To evaluate interference effect of non-AMA1 IgG, delta % inhibition was calculated as follows:

Delta % inhibition  =  (% inhibition of the US-total IgG alone) – (% inhibition of a mixture of a non-AMA1 IgG and US-total IgG).

Data were analyzed using Prism 5 (GraphPad Software, Inc., CA, USA) and p values less than 0.05 were considered significant.

## Results

### Characteristics of AMA1-specific and non-AMA1 IgGs from Malian children

We began with the affinity purification of AMA1-specific and non-AMA1 IgGs in order to evaluate the growth-inhibitory activity of the AMA1-specific fractions and to assess the possibility of interfering antibodies in the non-AMA1 IgGs from the Malian children. The antibody levels of each total IgG pool and non-AMA1(3D7)/AMA1(FVO) IgG were measured by ELISA and the results are shown in Table 1. The non-AMA1 IgGs had less than 10% of AMA1 antibody levels compared to the corresponding original total IgG pools. These results showed that the AMA1-specific purification method used in this study was efficient.

**Figure 1:**
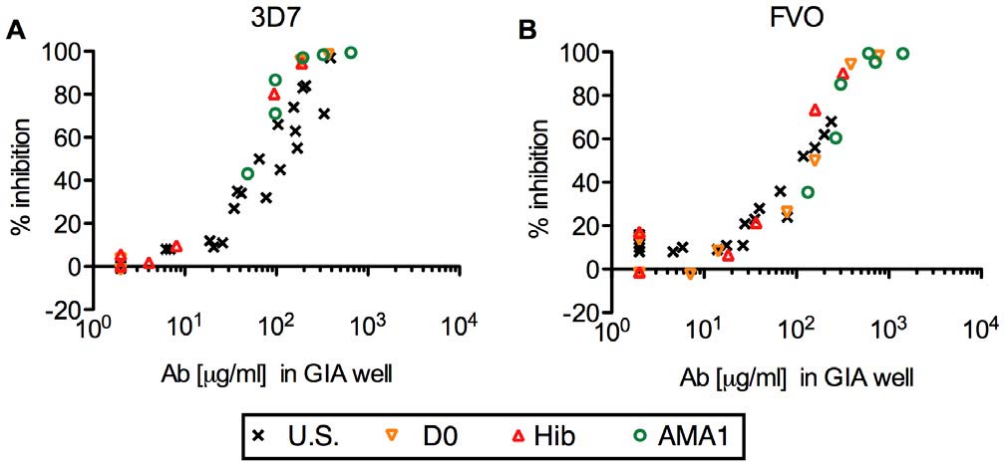
Strong correlation between anti-AMA1 antibody level and the growth-inhibitory activity in AMA1-specific IgGs. Anti-AMA1(3D7) (A) or anti-AMA1(FVO) (B) antibody levels (µg/ml) in the GIA well (*x*-axis) are plotted against % inhibition (*y*-axis) of *P. falciparum* 3D7 (A) or FVO (B) parasites. Each AMA1-specific IgG was tested at three (for U.S. IgGs) or two (for Mali IgGs) different concentrations. All responses below the limit of detection in ELISA were assigned a value of 2 µg/ml for the analysis.

We then investigated the biological activity of the AMA1-specific IgGs from U.S. vaccinees and Malian children by GIA (Figure 1). As in our previous study [27], the AMA1-specific IgGs from U.S. vaccinees showed a significant correlation between antibody level and percent inhibition when they were tested against the homologous strain of parasites (Spearman rank correlation, p<0.001, ρ_s_  = 0.96, 95% confidence interval (CI) 0.91–0.99 for 3D7; p<0.001, ρ_s_  = 0.83, 95% CI 0.60–0.94, for FVO) and the relationship followed a symmetrical sigmoid curve when the antibody levels were log transformed (r^2^ = 0.93 for 3D7 and 0.82 for FVO). The AMA1-specific IgGs from Malian children showed almost the same level of growth-inhibitory activity at the same level of antibody in the GIA well, regardless of which pools of total IgG were tested. When the data from all AMA1-specific IgGs from Malian children were combined, there were significant correlations between antibody levels by ELISA and percent inhibition in GIA (for 3D7, p<0.001, ρ_s_  = 0.97, 95% CI 0.92–0.99; for FVO, p<0.001, ρ_s_  = 0.94, 95% CI 0.86–0.98), and each relationship followed a symmetrical sigmoid curve (r^2^ = 0.99 for 3D7, 0.94 for FVO). These results indicate that there was no obvious difference in biological activity of antibodies among AMA1-specific IgGs induced by an AMA1 vaccination (IgGs from U.S. vaccinees), by natural infection (IgGs from D0-1, 2, 3 & 4 and Hib-1, 2 & 3 pools) and by both natural infection and vaccination (IgGs from AMA1-1, 2 & 3 pools).

**Figure 2:**
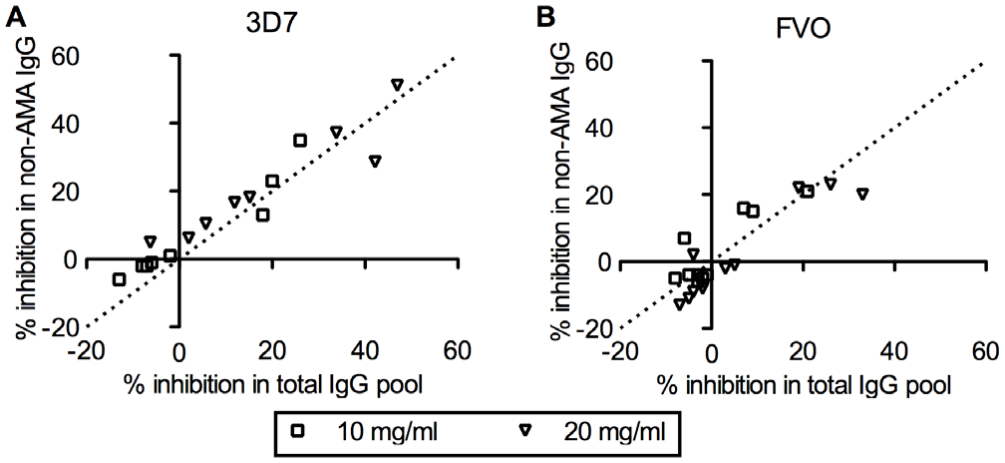
Non-AMA1 IgGs show comparable levels of growth-inhibitory activity as the original total IgG pools. Non-AMA1(3D7) (A) or non-AMA1(FVO) IgGs were tested against *P. falciparum* 3D7 (A) or FVO (B) parasites at 10 or 20 mg/ml in GIA well. Percent inhibition of non-AMA1 IgG (*y*-axis) is plotted against the % inhibition of the original total IgG pool (*x*-axis). The dotted line represents y = x line.

We next evaluated the activity of non-AMA1 IgGs from Malian children by GIA at two different concentrations in GIA well using homologous strain of parasites (i.e., non-AMA1(3D7) IgGs were tested against 3D7 strain of parasites and non-AMA1(FVO) IgG with FVO parasites). The % inhibition of non-AMA1 IgG was plotted against that of the original total IgG pool (Figure 2). The slope of the best-fit line is 0.97 (95%CI: 0.81–1.14) for the 3D7 data set and 0.87 (95%CI: 0.64–1.10) for FVO. Since the slope was not significantly different from 1, it showed that the depletion of AMA1-specific IgGs from the total IgG did not materially change growth-inhibitory activity, even though AMA1-specific IgGs from the same total IgG pools also showed activity (Figure 1).

### Non-AMA1 IgGs interfere with AMA1 IgG activity in GIA

**Figure 3:**
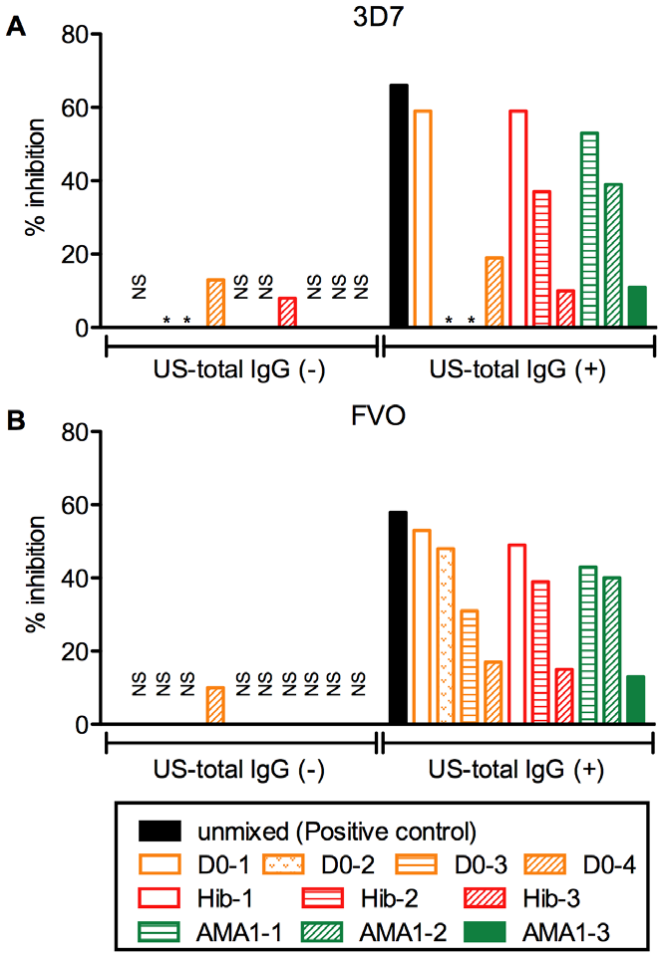
Non-AMA1 IgGs from Malian children reduce the growth-inhibitory activity of US-total IgG. (A) Four mg/ml of non-AMA1(3D7) IgGs were tested either by themselves (left side) or with US-total IgG (right side) using *P. falciparum* 3D7 parasites. The US-total IgG was tested alone as a positive control (black bar) (B). Similar study was performed using non-AMA(FVO) IgGs and *P. falciparum* FVO parasites. NS: % inhibition was in the range of ±5%. *: no non-AMA1 IgG was made in this study.

**Figure 4:**
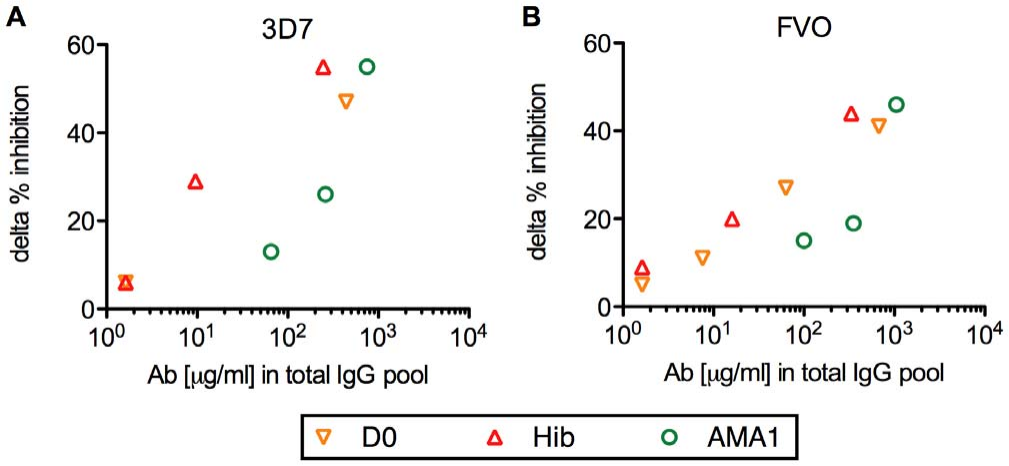
The correlation between anti-AMA1 antibody levels in the original total IgG pool and the interference effect of the corresponding non-AMA1 IgG. Anti-AMA1(3D7) (A) or anti-AMA1(FVO) (B) antibody levels (µg/ml) in the original total IgG pools (*x*-axis) are plotted against delta % inhibition of non-AMA1 IgGs (*y*-axis) tested with *P. falciparum* 3D7 (A) or FVO (B) parasites. Delta % inhibition of each non-AMA1 IgG was calculated using the data presented in Figure 3 as follows: delta % inhibition  =  (% inhibition of the US-total IgG alone (black bar in Figure 3)) - (% inhibition of a mixture of the non-AMA1 IgG and US-total IgG).

To assess interference effect in the non-AMA1 IgGs, non-AMA1 IgGs were tested by GIA either by themselves or in the presence of total IgG from a U.S. vaccinee (US-total IgG) against homologous strain of parasites (i.e., non-AMA1(3D7) IgGs were tested with or without US-total IgG using 3D7 strain of parasites in GIA, and non-AMA1(FVO) IgGs were similarly tested using FVO parasites). The US-total IgG was also tested alone as a positive control (black bar in Figure 3). The non-AMA1 IgGs showed less than 20 % inhibition at 4 mg/ml (Figure 3). The mixtures of non-AMA1 IgGs and US-total IgG displayed lower inhibition for both 3D7 and FVO parasites compared to the US-total IgG alone. To evaluate the strength of the interference effect of each non-AMA1 IgG, the difference between US-total IgG alone and the mixture was calculated (delta % inhibition). As shown in Figure 4, the non-AMA1 IgGs purified from total IgG pools with higher AMA1 antibody levels showed greater interference (larger delta % inhibition) than those from total IgG pools with lower titer. When all of the data were combined, there was a significant correlation between the AMA1 antibody level in the original total IgG pool and the interference effect of non-AMA1 IgG (Spearman rank correlation, p = 0.021, ρ_s_  = 0.80 for 3D7; p = 0.003, ρ_s_  = 0.82 for FVO).

**Figure 5:**
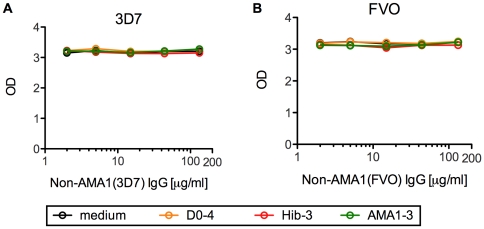
Competition ELISA with US-total IgG and non-AMA1 IgGs. (A) A fixed amount of US-total IgG, which gives approximately O.D. value of 3, was mixed with 3-fold dilutions of non-AMA1(3D7) IgGs from Malian children. The mixtures were tested by ELISA using an AMA1(3D7)-coated plate and the O.D. values are shown. (B) Similar study was performed using non-AMA(FVO) IgGs and an AMA1(FVO)-coated ELISA plate.

To test whether the interfering effect of non-AMA1 IgGs was due to the blocking of binding between AMA1 antigen and anti-AMA1 antibody, competition ELISA was performed. A fixed amount of US-total IgG was mixed with serially diluted non-AMA1 IgGs which showed a higher interference effect in Figure 3 (i.e., D0-4, Hib-3 and AMA1-3). The mixtures were applied to AMA1(3D7) or AMA1(FVO)-coated ELISA plates as primary antibodies and the amount of antibodies which bound to AMA1 protein was measured. The mixture with 14 µg/ml of non-AMA1(3D7) IgG in this ELISA using AMA1(3D7)-coated plates had the same ratio of US-total IgG and non-AMA1(3D7) IgG as tested in Figure 3 by GIA using 3D7 strain of parasites, and 11 µg/ml of non-AMA1(FVO) IgG for FVO. As shown in Figure 5, none of the non-AMA1 IgGs tested blocked binding of anti-AMA1 antibody in US-total IgG to the ELISA plates. The same assay was conducted using total IgG from another U.S. vaccinee and also showed no competition (data not shown). Thus it does not appear that the interfering effect is due to direct inhibition of the anti-AMA1 antibodies binding to the plate antigen.

## Discussion

In the present study, we have shown that the non-AMA1 IgGs from Malian children interfere with the growth-inhibitory activity of anti-AMA1 antibodies obtained from malaria naïve U.S. volunteers vaccinated with AMA1 (US-total IgG). Interestingly, the interference effect of non-AMA1 IgG from total IgG pools with higher titers of AMA1 was higher than those from total IgG pools with lower titers. However, while the non-AMA1 IgGs showed this interference in GIA, they did not block binding of anti-AMA1 antibodies in US-total IgG to AMA1 protein in a competition ELISA.

In the case of viruses, such as hepatitis C virus (HCV) or human immunodeficiency virus (HIV), there are studies showing either monoclonal or polyclonal antibodies can interfere with the effects of other monoclonal or polyclonal antibodies in a neutralizing assay [31]-[35]. In these studies, the antibodies recognize different epitopes on the same antigen and the mechanism of the interference may be explained by steric blocking [36] and/or conformational rearrangement induced by the non-neutralizing antibody. A similar phenomenon has been reported with the *P. falciparum* Merozoite Surface Protein 1 (MSP1), another blood-stage vaccine candidate. In the case of MSP1, a proportion of anti-MSP1 antibodies called “blocking” antibodies, which are found in people living in malaria endemic areas, competes with an anti-MSP1 monoclonal antibody capable of inhibiting merozoite invasion of erythrocytes in vitro, as judged by a competition ELISA and by an MSP1 processing assay [37]–[39]. However, it is not clear whether such “blocking” antibodies interfere with the activity of anti-MSP1 antibody, especially the activity of polyclonal antibodies, in a biological assay, such as GIA. It has been reported that human “blocking” antibodies interfere with the invasion-inhibitory activity of mouse anti-MSP1 monoclonal antibody (mAb) 12.8, but the same human antibodies did not block the biological activity of another inhibitory mAb 12.10 [37]. To our knowledge, non-AMA1 IgG is the only reported antibodies which have been shown to interfere with the growth-inhibitory activity induced by human antigen-specific polyclonal antibodies. However, it is conceivable that similar functional interference effect exists in antibodies against other malarial antigens, including MSP1.

In contrast to the cases of HCV, HIV or MSP1, the non-AMA1 IgGs tested in this study showed negligible level of binding to the AMA1 protein as judged by ELISA (Table 1). In addition, the non-AMA1 IgGs did not compete with US-total IgG in a competition ELISA (Figure 5). We tested up to ∼10 times greater ratio of non-AMA1 IgG to US-total IgG compared with the ratio tested in GIA (Figure 3) where non-AMA1 IgG showed this interference effect, but still there was no competition observed. Since the non-AMA1 IgGs had some residual anti-AMA1 antibodies in the final preparation, one may consider the possibility that the residual antibody in the non-AMA1 IgGs masked the competition effect. However, we think this is not the case. When non-AMA1 IgGs were tested at 11–14 µg/ml (which gave the same ratio of non-AMA1 IgG and US-total IgG as tested in Figure 3), the amount of anti-AMA1 antibody coming from the non-AMA1 IgG was less than 5% compared to the one from US-total IgG. Even at the highest concentration of non-AMA1 IgG tested (i.e., 133 µg/ml), anti-AMA1 antibody from non-AMA1 IgG was less than 30% compared to the one from US-total IgG. Therefore, we concluded the competition effect of non-AMA1 was not obvious if any. These results suggest that either binding between AMA1 protein and non-AMA1 IgGs was too weak to detect by conventional ELISA and/or the mechanism of interference by non-AMA1 IgG is an indirect effect. Not only the function of AMA1 during the invasion process, but also the mechanism of invasion inhibition by anti-AMA1 antibodies is still controversial. Some studies show that a rabbit polyclonal growth-inhibitory anti-AMA1 antibody disrupts proteolytic processing of AMA1 [40], [41] and other studies with inhibitory mAb or peptide show that they block complex formation of AMA1 and rhoptry neck proteins RON2, RON4 and RON5 [42], [43]. In our previous study [27], we have shown that the interfering activity was due to the malaria-specific IgGs in the non-AMA1 IgGs population. However, because the growth-inhibitory activity of non-AMA1 IgGs in the previous study was high (the IgGs were collected mainly from Malian adults), we couldn't measure the strength of the interference effect. In this study with IgGs from Malian children, we could not perform malaria-extract-specific IgG purification from the non-AMA1 IgGs because of the limited quantity of blood samples available. On the other hand, as the intrinsic growth-inhibitory activity of non-AMA1 IgGs from Malian children is relatively low, we can measure the strength of the interference effect. We found that the interference effect of non-AMA1 IgG from total IgG pools with higher AMA1 titers was greater than that from total IgG pools with lower AMA1 titers. We believe the higher AMA1 titer reflects more malaria exposure, because the Malian children with higher AMA1 titer also display higher titers against other malaria antigens such as MSP1 (our unpublished observation). Therefore, the correlation between anti-AMA1 antibody levels in the original total IgG pool and the strength of interference effect in non-AMA1 IgG suggests that the interference effect is due to antibody against a malaria antigen other than AMA1. If the interference mechanism of the non-AMA1 IgGs is indirect, as the ELISA and competition ELISA results suggest, it is possible that the non-AMA1 IgGs may bind to RON2, RON4 and/or RON5 first and then may block the ability of the anti-AMA1 growth-inhibitory antibody to bind to the critical site of complex formation. Further studies are required to reveal the mechanism of the interference IgGs, and such studies may enhance our understanding of the invasion-inhibition mechanism by human anti-AMA1 polyclonal antibodies.

In the mixture experiment (Figure 3), we used non-AMA1 IgGs at 4 mg/ml, while the physiological concentration of IgG in human serum is 10–20 mg/ml. In the previous study where we prepared non-AMA1 IgGs from Malian adults, 4 and 0.4 mg/ml of non-AMA1 IgGs were tested. However, 0.4 mg/ml of non-AMA1 IgGs did not show a clear interference effect [27]. The result suggests that a certain level of non-AMA1 IgGs is needed to detect interference effect in this assay. On the other hand, if we use non-AMA1 IgGs at 10 or 20 mg/ml, several of them show >20% inhibition by themselves (Figure 2), so that it is difficult to calculate the interference effect, as the mixture cannot show lower inhibition than non-AMA1 IgG alone. Therefore, we decided to use the same 4 mg/ml concentration as the previous study [27]. The concentration of AMA1-specific IgG in the US-total IgG used for the mixture experiment was at 133 (for 3D7 parasites) or 202 (for FVO) µg/ml, and median level of AMA1-specific antibody in Malian children after immunization was 111.6 µg/ml [28]. Therefore, it is reasonable to assume than non-AMA1 IgGs interfere with growth-inhibitory activity of AMA1-specific IgG at physiological ratio (i.e., mix 10–20 mg/ml of non-AMA1 IgGs with 100 µg/ml AMA1-specific IgG).

This study clearly shows that there are interference IgGs in the 2–3 year old children who are the main targets of a blood-stage vaccine. From a vaccine development point of view, one of the other concerns is whether the AMA1 vaccination by itself induces such interfering IgGs. In our previous study, we did not detect any interference effect of non-AMA1 IgG from U.S. vaccinees [27]. That study suggests that the AMA1 vaccination per se is unlikely to induce the interference IgG at least in a malaria naïve population. In this study, the volume of plasma from each Malian child was too small to make sufficient amounts of AMA1-specific and/or non-AMA1 IgGs for experiments. Even if we collected a larger amount of plasma from each individual child, it is practically impossible to differentiate AMA1-specific and/or non-AMA1 IgG induced by the vaccination from those induced by a natural infection in children living in a malaria endemic area. For that reason, it is difficult to exclude the possibility that AMA1 vaccination induces interfering IgGs in the target population. However, as shown in Figure 4, non-AMA1 IgGs from children immunized with the AMA1 vaccine showed the same (AMA1-3) or less (AMA1-1 and AMA1-2) interference than those from children without AMA1 vaccination with the same AMA1 levels in the original total IgG pools. Therefore, there is no evidence to suggest that AMA1 vaccination induced more interfering IgGs in this study, rather the data from AMA1-1 and AMA1-2 indicates that the interference antibody may be induced by natural infection, not by the AMA1 vaccination.

AMA1 is a highly polymorphic protein [44], [45] and it is obvious that polymorphism is one of the major obstacles to make a broadly effective vaccine [11]. Indeed, when AMA1 vaccines are administered in malaria naïve individuals, the anti-AMA1 antibodies induced by vaccination show growth-inhibitory activity against the homologous strain of parasites, but weaker or no activity against heterologous strains of parasites, regardless of adjuvant used [16], [19], [22]. In this study, we investigated both 3D7 and FVO strains of parasites and the results demonstrate that the interference effect occurs in both allelic forms of AMA1. However, we did not test the heterologous combination, e.g., a mixture of non-AMA1 (3D7) IgGs and US-total IgG tested against FVO strain of parasites. Because the non-AMA1(3D7) IgGs still had anti-AMA1(FVO)-allele-specific antibody in the preparation, which was confirmed by ELISA and GIA (data not shown), it is very difficult to interpret the result of mixture experiments with FVO parasites. We tested a tandem purification method (i.e., total IgGs were applied onto an AMA1(3D7) purification column and an AMA1(FVO) column sequentially) using rabbit anti-AMA1 antibodies, but there were technical problems with completely depleting anti-AMA1(3D7) and anti-AMA1(FVO)-allele-specific antibodies from the total IgGs (data not shown). Further studies could be conducted to test the cross-reactivity of the interfering antibodies if enough volume of starting blood materials is available.

One of the major questions in malaria vaccine development is what assay can serve as a surrogate for clinical protection. At this stage, there is no immunological assay proven to be correlated with clinical protection. As noted above, anti-AMA1 antibodies induced both by a malaria infection [26], [27] and by an AMA1 immunization in malaria naïve individuals [14], [16], [19], [21]–[23] show growth-inhibitory activity, and a recent clinical challenge trial with AMA1 vaccine in a malaria naïve population has shown that there is a significant correlation between in vivo parasite multiplication-rate and growth-inhibitory activity measured by in vitro GIA in vaccine recipients (our unpublished observation). In addition, some epidemiological studies have shown that the total growth-inhibitory activity (or invasion-inhibition activity) before the malaria transmission season is significantly associated with a subsequent malaria risk [46], [47]. However, other epidemiological studies have not shown such associations [48], [49]. Therefore, one may dispute the usage of this assay for vaccine development. These epidemiological studies did not test the specificity of antibodies. In addition, interpretation of growth-inhibitory activity of samples from epidemiological studies for a specific antigen is not straightforward. As shown in Figure 1, when anti-AMA1-specific IgGs were separated from total IgG pools of Malian children, they showed similar activity as anti-AMA1 IgG from U.S. vaccinees. However, the AMA1-depleted IgG, i.e., non-AMA1 IgG, displayed the same level of inhibition as the original total IgG pool (Figure 2). We also observed the same phenomenon in our previous study [27] where we separated non-AMA1 IgG from Malian adults' total IgGs. In addition, another of our studies [28] showed that pre-incubation of Malian children's total IgGs with AMA1 protein did not reduce growth-inhibitory activity induced by natural infection (even though the total IgGs had higher level of AMA1 titer), while the pre-incubation did diminish vaccine-induced activity almost completely. All of the data indicate that the overall growth-inhibitory activity induced by a natural infection is not simply the sum of growth-inhibitory activity of individual antibodies. Our preliminary study shows that there is no additive effect of growth-inhibitory activity between rabbit anti-AMA1 antibody and anti-MSP1 antibody (our unpublished observation). Furthermore, it is possible that malaria infection induces not only growth-inhibitory antibodies, but also interfering antibodies in humans, at least in the case of AMA1, as shown in this study. While it is difficult to prove whether such non-additive (and/or interference) effects really occur in vivo, or whether this is just a limitation of the in vitro assay, in either case the growth-inhibitory activity of antibodies from a malaria endemic area for a specific antigen should be interpreted with caution. However, the GIA is the only functional assay widely used for AMA1-based vaccine development. If the growth-inhibitory activity measured by the GIA reflects some mechanism of protection in vivo, the results of this study suggest that pre-existing anti-malaria immunity may modulate the efficacy of the AMA1 vaccine. At a minimum, we believe it is extremely important to take these findings into account in evaluating immunogenicity of AMA1-based vaccines when a study is conducted in populations exposed to malaria.

## References

[1] (2009).

[2] Cohen S, Mc GI, Carrington S (1961). Gamma-globulin and acquired immunity to human malaria.. Nature.

[3] Kilama W, Ntoumi F (2009). Malaria: a research agenda for the eradication era.. Lancet.

[4] Triglia T, Healer J, Caruana SR, Hodder AN, Anders RF (2000). Apical membrane antigen 1 plays a central role in erythrocyte invasion by *Plasmodium* species.. Mol Microbiol.

[5] Fraser TS, Kappe SH, Narum DL, VanBuskirk KM, Adams JH (2001). Erythrocyte-binding activity of *Plasmodium yoelii* apical membrane antigen-1 expressed on the surface of transfected COS-7 cells.. Mol Biochem Parasitol.

[6] Urquiza M, Suarez JE, Cardenas C, Lopez R, Puentes A (2000). *Plasmodium falciparum* AMA-1 erythrocyte binding peptides implicate AMA-1 as erythrocyte binding protein.. Vaccine.

[7] Kato K, Mayer DC, Singh S, Reid M, Miller LH (2005). Domain III of *Plasmodium falciparum* apical membrane antigen 1 binds to the erythrocyte membrane protein Kx.. Proc Natl Acad Sci U S A.

[8] Mitchell GH, Thomas AW, Margos G, Dluzewski AR, Bannister LH (2004). Apical membrane antigen 1, a major malaria vaccine candidate, mediates the close attachment of invasive merozoites to host red blood cells.. Infect Immun.

[9] Treeck M, Zacherl S, Herrmann S, Cabrera A, Kono M (2009). Functional analysis of the leading malaria vaccine candidate AMA-1 reveals an essential role for the cytoplasmic domain in the invasion process.. PLoS pathog.

[10] Silvie O, Franetich JF, Charrin S, Mueller MS, Siau A (2004). A Role for Apical Membrane Antigen 1 during Invasion of Hepatocytes by *Plasmodium falciparum* Sporozoites.. J Biol Chem.

[11] Remarque EJ, Faber BW, Kocken CH, Thomas AW (2008). Apical membrane antigen 1: a malaria vaccine candidate in review.. Trends Parasitol.

[12] Stowers AW, Kennedy MC, Keegan BP, Saul A, Long CA (2002). Vaccination of monkeys with recombinant *Plasmodium falciparum* apical membrane antigen 1 confers protection against blood-stage malaria.. Infect Immun.

[13] Dutta S, Sullivan JS, Grady KK, Haynes JD, Komisar J (2009). High antibody titer against apical membrane antigen-1 is required to protect against malaria in the Aotus model.. PLoS ONE.

[14] Malkin EM, Diemert DJ, McArthur JH, Perreault JR, Miles AP (2005). Phase 1 clinical trial of apical membrane antigen 1: an asexual blood-stage vaccine for *Plasmodium falciparum* malaria.. Infect Immun.

[15] Dicko A, Diemert DJ, Sagara I, Sogoba M, Niambele MB (2007). Impact of a *Plasmodium falciparum* AMA1 Vaccine on Antibody Responses in Adult Malians.. PLoS ONE.

[16] Mullen GE, Ellis RD, Miura K, Malkin E, Nolan C (2008). Phase 1 trial of AMA1-C1/Alhydrogel plus CPG 7909: an asexual blood-stage vaccine for *Plasmodium falciparum* malaria.. PLoS ONE.

[17] Sagara I, Ellis RD, Dicko A, Niambele MB, Kamate B (2009). A randomized and controlled Phase 1 study of the safety and immunogenicity of the AMA1-C1/Alhydrogel((R))+CPG 7909 vaccine for *Plasmodium falciparum* malaria in semi-immune Malian adults.. Vaccine.

[18] Dicko A, Sagara I, Ellis RD, Miura K, Guindo O (2008). Phase 1 Study of a Combination AMA1 Blood Stage Malaria Vaccine in Malian Children.. PLoS ONE.

[19] Polhemus ME, Magill AJ, Cummings JF, Kester KE, Ockenhouse CF (2007). Phase I dose escalation safety and immunogenicity trial of *Plasmodium falciparum* apical membrane protein (AMA-1) FMP2.1, adjuvanted with AS02A, in malaria-naive adults at the Walter Reed Army Institute of Research.. Vaccine.

[20] Thera MA, Doumbo OK, Coulibaly D, Diallo DA, Kone AK (2008). Safety and immunogenicity of an AMA-1 malaria vaccine in Malian adults: results of a phase 1 randomized controlled trial.. PLoS ONE.

[21] Roestenberg M, Remarque E, de Jonge E, Hermsen R, Blythman H (2008). Safety and immunogenicity of a recombinant Plasmodium falciparum AMA1 malaria vaccine adjuvanted with Alhydrogel, Montanide ISA 720 or AS02.. PLoS ONE.

[22] Spring MD, Cummings JF, Ockenhouse CF, Dutta S, Reidler R (2009). Phase 1/2a study of the malaria vaccine candidate apical membrane antigen-1 (AMA-1) administered in adjuvant system AS01B or AS02A.. PLoS ONE.

[23] Ellis RD, Mullen GE, Pierce M, Martin LB, Miura K (2009). A Phase 1 study of the blood-stage malaria vaccine candidate AMA1-C1/Alhydrogel((R)) with CPG 7909, using two different formulations and dosing intervals.. Vaccine.

[24] Thera MA, Doumbo OK, Coulibaly D, Laurens MB, Kone AK (2010). Safety and Immunogenicity of an AMA1 Malaria Vaccine in Malian Children: Results of a Phase 1 Randomized Controlled Trial.. PLoS ONE.

[25] Sagara I, Dicko A, Ellis RD, Fay MP, Diawara SI (2009). A randomized controlled phase 2 trial of the blood stage AMA1-C1/Alhydrogel malaria vaccine in children in Mali.. Vaccine.

[26] Hodder AN, Crewther PE, Anders RF (2001). Specificity of the protective antibody response to apical membrane antigen 1.. Infect Immun.

[27] Miura K, Zhou H, Moretz SE, Diouf A, Thera MA (2008). Comparison of biological activity of human anti-apical membrane antigen-1 antibodies induced by natural infection and vaccination.. J Immunol.

[28] Miura K, Zhou H, Diouf A, Tullo G, Moretz SE (2011). Immunological responses against *Plasmodium falciparum* Apical Membrane Antigen 1 vaccines vary depending on the population immunized. Vaccine..

[29] Miura K, Orcutt AC, Muratova OV, Miller LH, Saul A (2008). Development and characterization of a standardized ELISA including a reference serum on each plate to detect antibodies induced by experimental malaria vaccines.. Vaccine.

[30] Miura K, Zhou H, Diouf A, Moretz SE, Fay MP (2009). Anti-apical-membrane-antigen-1 antibody is more effective than anti-42-kilodalton-merozoite-surface-protein-1 antibody in inhibiting *Plasmodium falciparum* growth, as determined by the in vitro growth inhibition assay.. Clin Vaccine Immunol.

[31] Taylor HP, Dimmock NJ (1994). Competitive binding of neutralizing monoclonal and polyclonal IgG to the HA of influenza A virions in solution: only one IgG molecule is bound per HA trimer regardless of the specificity of the competitor.. Virology.

[32] Verrier F, Nadas A, Gorny MK, Zolla-Pazner S (2001). Additive effects characterize the interaction of antibodies involved in neutralization of the primary dualtropic human immunodeficiency virus type 1 isolate 89.6.. J Virol.

[33] Zhang P, Wu CG, Mihalik K, Virata-Theimer ML, Yu MY (2007). Hepatitis C virus epitope-specific neutralizing antibodies in Igs prepared from human plasma.. Proc Natl Acad Sci U S A.

[34] Zhang P, Zhong L, Struble EB, Watanabe H, Kachko A (2009). Depletion of interfering antibodies in chronic hepatitis C patients and vaccinated chimpanzees reveals broad cross-genotype neutralizing activity.. Proc Natl Acad Sci U S A.

[35] Zhong L, Haynes L, Struble EB, Tamin A, Virata-Theimer ML (2009). Antibody-mediated synergy and interference in the neutralization of SARS-CoV at an epitope cluster on the spike protein.. Biochem Biophys Res Commun.

[36] Massey RJ, Schochetman G (1981). Viral epitopes and monoclonal antibodies: isolation of blocking antibodies that inhibit virus neutralization.. Science.

[37] Guevara Patino JA, Holder AA, McBride JS, Blackman MJ (1997). Antibodies that inhibit malaria merozoite surface protein-1 processing and erythrocyte invasion are blocked by naturally acquired human antibodies.. J Exp Med.

[38] Nwuba RI, Sodeinde O, Anumudu CI, Omosun YO, Odaibo AB (2002). The human immune response to *Plasmodium falciparum* includes both antibodies that inhibit merozoite surface protein 1 secondary processing and blocking antibodies.. Infect Immun.

[39] Okech BA, Corran PH, Todd J, Joynson-Hicks A, Uthaipibull C (2004). Fine specificity of serum antibodies to *Plasmodium falciparum* merozoite surface protein, PfMSP-1(19), predicts protection from malaria infection and high-density parasitemia.. Infect Immun.

[40] Dutta S, Haynes JD, Moch JK, Barbosa A, Lanar DE (2003). Invasion-inhibitory antibodies inhibit proteolytic processing of apical membrane antigen 1 of *Plasmodium falciparum* merozoites.. Proc Natl Acad Sci U S A.

[41] Dutta S, Haynes JD, Barbosa A, Ware LA, Snavely JD (2005). Mode of action of invasion-inhibitory antibodies directed against apical membrane antigen 1 of *Plasmodium falciparum*.. Infect Immun.

[42] Collins CR, Withers-Martinez C, Hackett F, Blackman MJ (2009). An inhibitory antibody blocks interactions between components of the malarial invasion machinery.. PLoS pathog.

[43] Richard D, Macraild CA, Riglar DT, Chan JA, Foley M (2010). Interaction between *Plasmodium falciparum* apical membrane antigen 1 and the Rhoptry neck protein complex defines a key step in the erythrocyte invasion process of malaria parasites.. J Biol Chem.

[44] Marshall VM, Zhang L, Anders RF, Coppel RL (1996). Diversity of the vaccine candidate AMA-1 of *Plasmodium falciparum*.. Mol Biochem Parasitol.

[45] Duan J, Mu J, Thera MA, Joy D, Kosakovsky Pond SL (2008). Population structure of the genes encoding the polymorphic *Plasmodium falciparum* apical membrane antigen 1: implications for vaccine design.. Proc Natl Acad Sci U S A.

[46] Crompton PD, Miura K, Traore B, Kayentao K, Ongoiba A (2010). In vitro growth-inhibitory activity and malaria risk in a cohort study in mali.. Infect Immun.

[47] Dent AE, Bergmann-Leitner ES, Wilson DW, Tisch DJ, Kimmel R (2008). Antibody-mediated growth inhibition of *Plasmodium falciparum*: relationship to age and protection from parasitemia in Kenyan children and adults.. PLoS ONE.

[48] Marsh K, Otoo L, Hayes RJ, Carson DC, Greenwood BM (1989). Antibodies to blood stage antigens of *Plasmodium falciparum* in rural Gambians and their relation to protection against infection.. Trans R Soc Trop Med Hyg.

[49] Perraut R, Marrama L, Diouf B, Sokhna C, Tall A (2005). Antibodies to the conserved C-terminal domain of the *Plasmodium falciparum* merozoite surface protein 1 and to the merozoite extract and their relationship with in vitro inhibitory antibodies and protection against clinical malaria in a Senegalese village.. J Infect Dis.

